# Aortic Baroreceptors Display Higher Mechanosensitivity than Carotid Baroreceptors

**DOI:** 10.3389/fphys.2016.00384

**Published:** 2016-08-31

**Authors:** Eva On-Chai Lau, Chun-Yin Lo, Yifei Yao, Arthur Fuk-Tat Mak, Liwen Jiang, Yu Huang, Xiaoqiang Yao

**Affiliations:** ^1^School of Biomedical Sciences, Li Ka Shing Institute of Health Sciences, Faculty of Medicine, The Chinese University of Hong KongHong Kong, Hong Kong; ^2^Division of Biomedical Engineering, The Chinese University of Hong KongHong Kong, Hong Kong; ^3^School of Life Sciences, The Chinese University of Hong KongHong Kong, Hong Kong

**Keywords:** aortic baroreceptor, carotid baroreceptor, mechano-sensitivity, electrophysiology, action potential

## Abstract

Arterial baroreceptors are mechanical sensors that detect blood pressure changes. It has long been suggested that the two arterial baroreceptors, aortic and carotid baroreceptors, have different pressure sensitivities. However, there is no consensus as to which of the arterial baroreceptors are more sensitive to changes in blood pressure. In the present study, we employed independent methods to compare the pressure sensitivity of the two arterial baroreceptors. Firstly, pressure-activated action potential firing was measured by whole-cell current clamp with a high-speed pressure clamp system in primary cultured baroreceptor neurons. The results show that aortic depressor neurons possessed a higher percentage of mechano-sensitive neurons. Furthermore, aortic baroreceptor neurons show a lower pressure threshold than that of carotid baroreceptor neurons. Secondly, uniaxial stretching of baroreceptor neurons, that mimics the forces exerted on blood vessels, elicited a larger increase in intracellular Ca^2+^ rise in aortic baroreceptor neurons than in carotid baroreceptor neurons. Thirdly, the pressure-induced action potential firing in the aortic depressor nerve recorded *in vivo* was also higher. The present study therefore provides for a basic physiological understanding on the pressure sensitivity of the two baroreceptor neurons and suggests that aortic baroreceptors have a higher pressure sensitivity than carotid baroreceptors.

## Introduction

Arterial baroreceptors serve as the frontline sensors to detect blood pressure changes in the blood stream. Changes in blood pressure stimulate the mechanosensitive nerve endings that are localized in the arterial walls. The mechanical force is transduced into electrical signals at the nerve terminals, resulting in pressure-dependent action potential firings in baroreceptor neurons. The nerve signals then propagate to the cardiovascular control center in the brainstem for baroreflex regulation of blood pressure (Levy MN, 2007). In addition, supra-medullary structures, including midbrain cuneiform nucleus and ventral medial prefrontal cortex, also have an inhibitory role in baroreflex regulation (Verberne et al., 1997).

**Figure 1:**
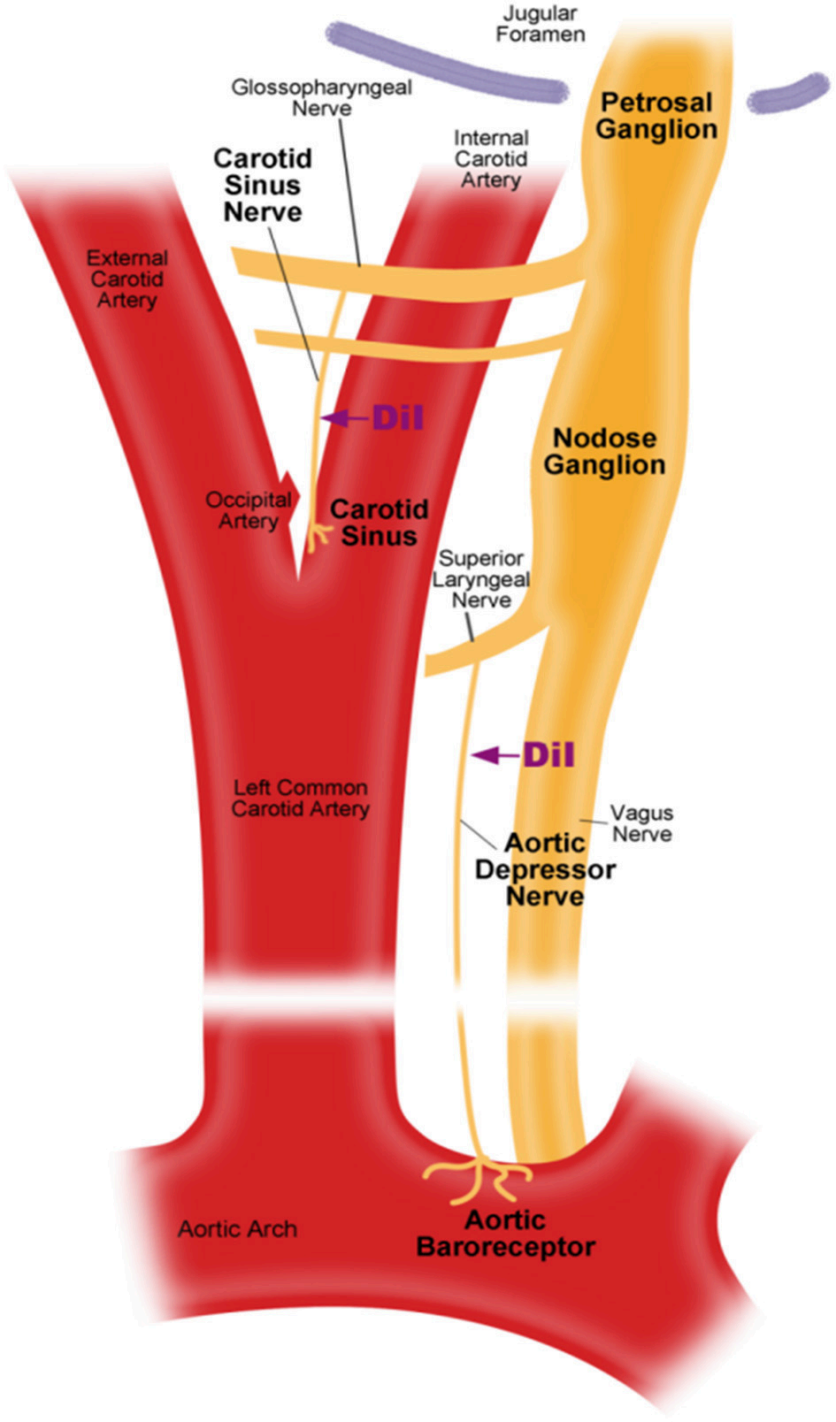
**Schematic diagram of aortic and carotid baroreceptor terminals and nerves**. Diagram illustrating the anatomical positions of aortic and carotid baroreceptors nerve terminals, their nerve fibers and somata regions. Aortic baroreceptor terminals are located in aortic arch. Its afferent nerve fiber is aortic depressor nerve. The soma is in the nodose ganglion (NG). Carotid baroreceptor is positioned in the internal carotid artery next to the carotid bifurcation. Its afferent nerve is carotid sinus nerve. The soma is located in the petrosal ganglion (PG).

There are two arterial baroreceptors, namely, the aortic baroreceptors and carotid baroreceptors, located in the adventitia layer of the aortic arch and carotid arteries, respectively. The aortic baroreceptors detect blood pressure in the aorta. The cell bodies (soma) of the aortic baroreceptors are located at the nodose ganglion (NG) (Figure 1). The nerve signals from the aortic baroreceptor nerve terminals are transmitted to the nodose ganglion through a sensory nerve named aortic depressor nerve (ADN) (Figure 1). Carotid baroreceptors detect the blood pressure in the carotid artery, which supplies blood to the brain. The nerve terminals of carotid baroreceptors are located bilaterally at the carotid artery bifurcations, close to the internal carotid artery. The nerve signals from the carotid baroreceptors travel along carotid sinus nerves (CSN) to their soma localized in the petrosal ganglion (PG). Petrosal ganglion protrudes beyond the jugular foramen (Figure 1) (McDonald, 1983; Shoukas et al., 1991; Vander et al., 1998; Sato et al., 1999; Donnelly and Rigual, 2000; Weijnen et al., 2000). Because aortic baroreceptors sense blood pressure in the aorta, which supplies blood to the whole systemic circulation, these baroreceptors are expected to play important functional role in the maintenance of overall systemic blood pressure. On the other hand, carotid baroreceptors detect the pressure of the blood that is being delivered to the brain. These baroreceptors may be more important for the maintenance of a stable cerebral blood pressure and cerebral blood flow.

There are conflicting reports as to which baroreceptors, aortic baroreceptors or carotid baroreceptors, are more sensitive to blood pressure change. Some researchers reported that the carotid baroreceptors are more sensitive to pressure (Donald and Edis, 1971; Fadel et al., 2003), whereas others believe that aortic baroreceptors are more sensitive to pressure (Glick and Covell, 1968; Pickering et al., 2008), and yet others have reported no difference in the pressure sensitivity between these two baroreceptors (Hainswor et al., 1970; James and Daly, 1970). However, almost all of these previous studies used only the baroreflex responses (such as heart rate and renal sympathetic nerve activity) as the end points for baroreceptor sensitivity assay. There is still no study which directly records and compares the pressure sensitivity of aortic and carotid baroreceptor neurons *per se*.

Here, we have used a high speed pressure clamp to alter the hydrostatic pressure inside of baroreceptor neurons and measured the corresponding electrophysiological responses of these neurons by whole-cell patch clamping. We also used a uniaxial stretch chamber to stretch the baroreceptor neurons and measured the corresponding changes in cytosolic Ca^2+^ in these neurons. In addition, for *in vivo* animal studies we measured the pressure-induced action potential firings in the aortic depressor and carotid sinus nerves, which are the sensory nerves of aortic and carotid baroreceptors respectively.

## Materials and methods

### Animals

All animal experiments were conducted under the authority of a license issued by the Government of the Hong Kong SAR and approval from the Animal Experimentation Ethics Committee, the Chinese University of Hong Kong. Male Sprague-Dawley (S/D) rats (180–200 g) were provided by Laboratory Animal Services Centre of the Chinese University of Hong Kong.

### Primary culture

S/D rats were anesthetized with pentobarbital sodium (100 mg/kg). The left cervical area was exposed by midline incision under sterilized condition. As shown in Figure 1, aortic depressor nerve or carotid sinus nerve was exposed and 2–3 mm of the nerve was carefully detached from surrounding tissues by blunt-dissection. Fluorescent lipophilic tracer 1,1-dioctadecyl-3,3,3′,3′-tetramethylindocarbocyanine (DiI) crystals (Invitrogen) were applied around the nerve and covered with Kwik-Sil (World Precision Instruments, USA). The incision was sutured afterward. The rats were allowed to recover for 5–7 days to allow DiI dye to diffuse retrogradely along the aortic depressor nerve or carotid sinus nerve to the somata located in nodose ganglion or petrosal ganglion respectively.

Nodose or petrosal ganglion neurons were isolated from DiI-labeled rats and cut into pieces in ice-cold EBSS. They were subjected to digestion for 1 h at 37°C with trypsin (1 mg/ml) and collagenase IA (1 mg/ml). Single neurons were dispersed by gentle trituration by Glass Pasteur pipette, followed by centrifugation. The neurons were resuspended and cultured in DMEM/F-12 media supplemented with 5% FBS, 1% antibiotic-antimycotic and 7S NGF (100 ng/ml). Cytosine arabinofuranoside (Ara-C; 10 μM) was included in the culture medium to inhibit the growth of dividing cells. The neurons were cultured for at least 3 days prior to experiments. For action potential recording, the neurons were freshly isolated and incubated in F12 at least 30 min before experiments. For all experiments, the cells were incubated on glass slides pre-coated with 0.1 mg/ml poly-L-lysine, except for the cell stretching experiments in which the silicone chambers were pre-coated with 2% gelatin.

### Patch clamp

Whole-cell current clamp recording were achieved by an EPC7 patch clamp amplifier (HEKA, Germany). Patch pipettes with resistance 3–5 MΩ were filled with pipette solution (in mM): 130 K-gluconate, 10 KCl, 2 MgCl_2_, 2 Na_2_ATP, 0.4 Tris GTP, 1 EGTA, 10 HEPES, pH 7.25-7.3 by KOH. Cells were bathed in the artificial cerebro-spinal fluid (ACSF) (in mM): 120 NaCl, 2 KCl, 1.2 MgSO_4_·7H_2_O, 1.2 KH_2_PO_4_, 26 NaHCO_3_, 2.5 CaCl_2,_ 11 glucose, equilibrated with carbogen (95% O_2_ and 5% CO_2_). In current clamp recording, the cells were held at its resting membrane potential. A high-speed pressure clamp system (HSPC-1, ALA Scientific Instruments, USA) was used to provide positive pressure to the cell through micropipette. Recordings were sampled at 50 kHz and filtered at 5 kHz. Data was analyzed with PulseFit (HEKA). All the experiments were performed at room temperature.

### Calcium imaging

Cytosolic Ca^2+^ measurement was performed as described elsewhere (Wong et al., 2010). Briefly, cells were loaded with 5 μM Fluo-4/AM for 40 min. Cytosolic Ca^2+^ measurement was performed with HEPES-buffered solution (in mM): 140 NaCl, 2.5 KCl, 1 MgCl_2_, 1 CaCl_2,_10 HEPES, 10 glucose, pH 7.4 by NaOH. Cytosolic Ca^2+^ change in response to uniaxial stretch was performed with STREX Cell Stretching System (ST-150, B-Bridge International, Inc.). The cells were stimulated by 10, 20, or 30% of unidirectional stretch for 1 s. Cytosolic Ca^2+^ response was measured at room temperature. The Fluo-4 fluorescence was recorded and analyzed by the FV1000 laser scanning confocal imaging system. Change in cytosolic Ca^2+^ fluorescence response was expressed as a ratio (F_x_/F_0_) of real-time fluorescence, where F_x_ is the real-time fluo-4/AM signal and F_0_ is the baseline fluo-4/AM signal before stretch.

### Nerve activity recording

Rats were anesthetized with 100 mg/kg pentobarbital sodium. The cervical area was exposed by midline incision. The right carotid artery was isolated and cannulated by a catheter that was connected to the pressure transducer (ML221, ADInstruments, USA) for blood pressure recording. The left femoral vein was cannulated for phenylephrine and SNP injection. The left aortic depressor nerve or carotid sinus nerve was connected to a bipolar silver electrode, followed by and an amplifier (Model 1700 Differential AC Amplifier, A-M Systems Inc., USA). Both the blood pressure and nerve activity were continuously recorded by Chart 5.0 (AD Instruments). The nerve activity was amplified 10,000 times and filtered at bandpass of 100–5000 Hz. Values of mean arterial pressure, heart rate and spike frequency in response to phenylephrine were analyzed by Chart 5.0 software. Change in spike frequency (spike frequency after phenylephrine—spike frequency before phenylephrine) in response to maximal blood pressure change (mean arterial blood pressure after phenylephrine—mean arterial blood pressure before phenylephrine) upon phenylephrine injection was calculated (Lau et al., 2016).

### Statistical analysis

Representative traces were plotted as time course traces. Data from all experiments were summarized into bar chart that was expressed in mean ± sem of individual experiments. Student's *t*-test was used for statistical analysis. Pairwise *t*-test was used where appropriate.

## Results

### Properties of aortic depressor neurons and carotid sinus neurons

**Figure 2:**
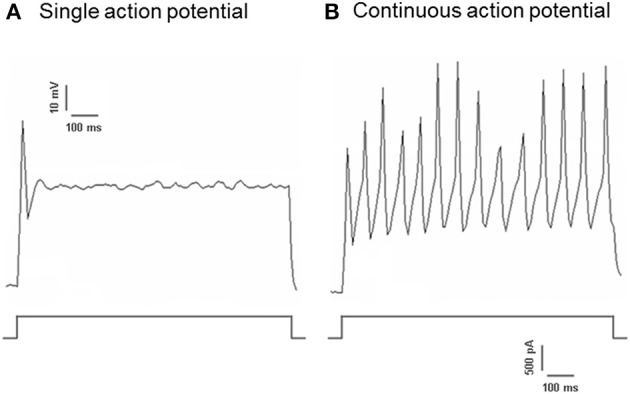
**Action potential recording from cultured aortic depressor and carotid sinus neurons**. Shown are the traces of action potentials that were recorded from two representative aortic depressor/ carotid sinus neurons upon a depolarizing current. **(A)** A representative neurons that fire only a single action potential upon the current stimulation; **(B)** A representative neurons that fire only continuous action potentials upon the current stimulation. Lower panel: protocol for current injection. Scale bar, horizontal, 100 ms, vertical, 10 mV (upper traces), and 500 pA (lower traces). *n* = 27 and *n* = 38 for aortic depressor and carotid sinus neurons respectively.

Dil was applied to either the aortic depressor nerve or carotid sinus nerve. Therefore, Dil-labeled neurons in nodose ganglion included the baroreceptor neurons innervating aortic arch and the chemoreceptor neurons innervating aortic body. On the other hand, Dil-labeled neurons in petrosal ganglion included the baroreceptor neurons innervating carotid sinus and the chemoreceptor neurons innervating the carotid body. We first investigated basic properties of these Dil-label neurons. DiI-labeled neurons in nodose and petrosal ganglion were cultured separately and subjected to a depolarizing electrical current as shown in Figure 2. Two types of responses were recorded, one responded with a single action potential and the other with continuous action potentials after the depolarizing current (Figure 2) (Belmonte and Gallego, 1983). These neurons were subsequently subjected to a hydrostatic pressure protocol in order to test their sensitivity to changes in hydrostatic pressure. A positive hydrostatic pressure ramp was applied intracellularly through a glass micropipette by the high-speed pressure clamp system (HSPC-1, ALA Scientific Instruments, USA) until action potentials were elicited. The results showed that 45% of aortic baroreceptor neurons fired continuous action potentials in response to the depolarizing electrical current (Table 1A). Among them, 91% (41%/45%) also responded to the hydrostatic pressure change and showed pressure-dependent action potential firings (Table 1A). The remaining 55% of aortic baroreceptor neurons only fired a single action potential in response to the depolarizing current (Table 1A), and among them, 40% (22%/55%) could respond to the pressure change (Table 1A). For the carotid baroreceptor neurons, 45% of the cells gave continuous action potentials when they were stimulated by the depolarizing current (Table 1B). Among them, 82% (37%/45%) were also sensitive to the pressure change (Table 1B). In addition, among the carotid baroreceptor neurons that only fired a single action potential in response to the depolarizing current, 24% (13%/55%) could respond to the pressure change (Table 1B).

**Table 1:** **Properties of aortic depressor and carotid sinus neurons**.

**(A) AORTIC DEPRESSOR NEURONS**	
Single action potential (55%)	Activated by hydrostatic pressure (22%)^*^
	Not activated by hydrostatic pressure (33%)
Continuous action potential (45%)	Activated by hydrostatic pressure (41%)^*^
	Not activated by hydrostatic pressure (4%)
**(B) CAROTID SINUS NEURONS**	
Single action potential (55%)	Activated by hydrostatic pressure (13%)^**^
	Not activated by hydrostatic pressure (42%)
Continuous action potential (45%)	Activated by hydrostatic pressure (37%)^**^
	Not activated by hydrostatic pressure (8%)

* *Percentage of aortic depressor neurons sensitive to hydrostatic pressure = 22% + 41% = 63%*.

** *Percentage of carotid sinus neurons sensitive to hydrostatic pressure = 13% + 37% = 50%. Summary shows the percentage of aortic depressor and carotid sinus neurons that gave different responses to depolarizing current and hydrostatic pressure. The primary cultured aortic depressor **(A)** or carotid sinus neurons **(B)** were stimulated by a depolarizing current and the resultant action potential pattern was recorded. These cells were subsequently exposed to a positive pressure transmitted to the inside of cells through micropipette using high-speed pressure clamp system. The resultant action potential pattern was recorded again. The responses of the neurons were classified into four categories: the cells eliciting single action potential upon current stimulation, further categorizing into activated by hydrostatic pressure or not activated by hydrostatic pressure; the cells eliciting continuous action potentials upon current stimulation, further categorizing into activated by hydrostatic pressure or not activated by hydrostatic pressure. n = 27 and n = 38 for aortic depressor and carotid sinus neurons respectively*.

Regardless of whether they fired a single action potentials or continuous action potentials, 63% of Dil-labeled aortic baroreceptor neurons were found to be sensitive to the pressure change, while only 50% of Dil-labeled carotid baroreceptor neurons were sensitive to the pressure change (Table 1).

### Stretch sensitivity of aortic and carotid baroreceptor neurons

**Figure 3:**
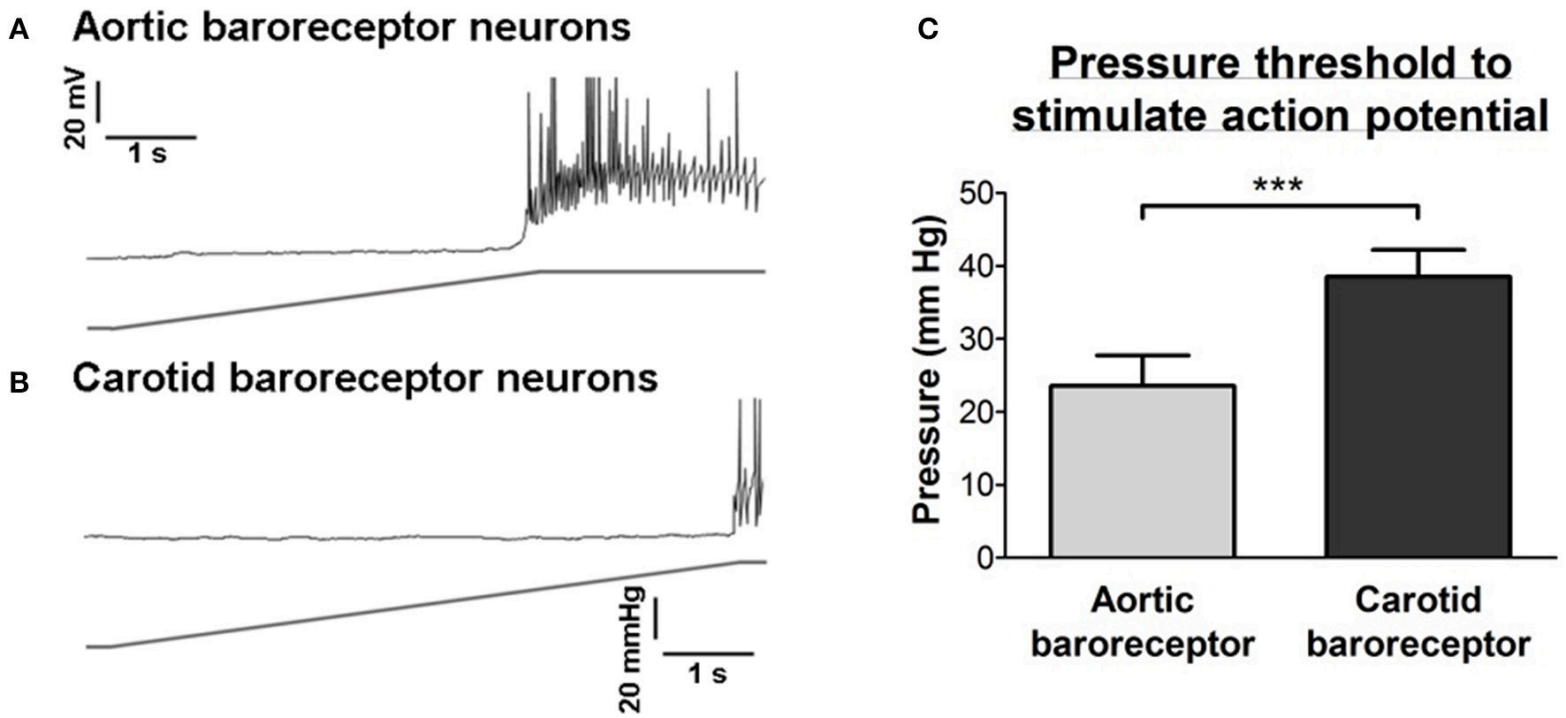
**Sensitivities of aortic and carotid baroreceptor neurons to hydrostatic pressure. (A,B)** Upper panel, representative time course traces illustrating the pressure-dependent action potential firings in aortic **(A)** and carotid **(B)** baroreceptor neurons. The pressure ramp (lower panel) was applied to the inside of cells through a micropipette using a high-speed pressure clamp system (HSPC). Scale bar horizontal, 1 s, vertical, 20 mV and 20 mmHg. **(C)** Summary showing the pressure threshold that initiates the action potential firings in aortic and carotid baroreceptor neurons. *n* = 13 for aortic baroreceptor neurons and *n* = 15 for carotid baroreceptor neurons. ^***^p < 0.01, by Student's *t*-test.

The pressure threshold for the action potential discharge was analyzed. As shown in Figure 3, aortic baroreceptor neurons had a lower pressure threshold (24 ± 4 mmHg) than that of the carotid baroreceptor neurons (39 ± 4 mmHg).

### Stretch-induced Ca^2+^ response in baroreceptor neurons

**Figure 4:**
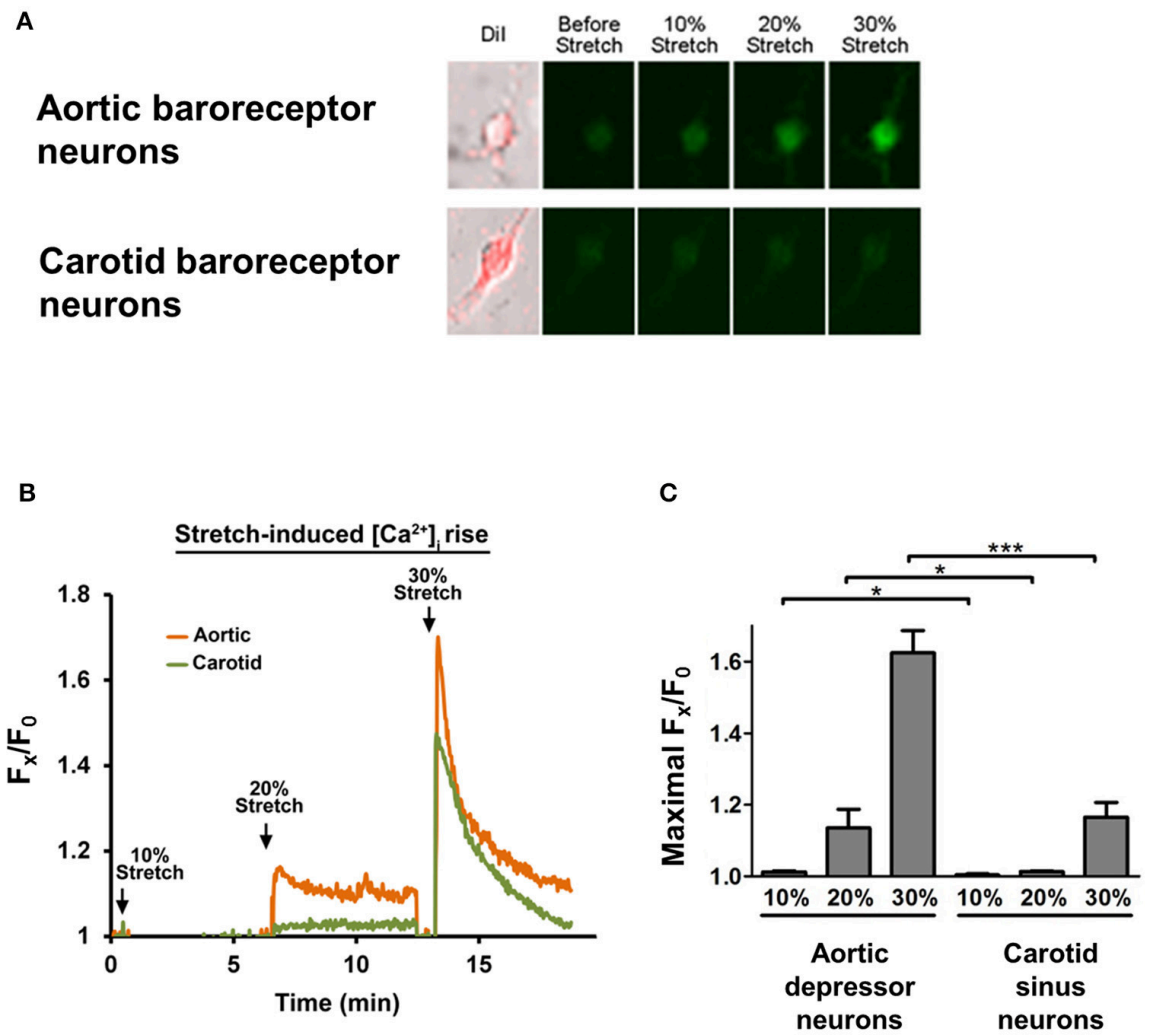
**Sensitivities of aortic depressor and carotid sinus neurons to uniaxial stretch**. Cells were loaded with Ca^2+^-sensitive fluorescent dye Fluo-4/AM. Shown are representative images **(A)** and time-course traces **(B)** of relative cytosolic Ca^2+^ rises in response to uniaxial stretch of 10, 20, and 30% of cell length in aortic depressor and carotid sinus neurons. **(C)** Summary of the maximal cytosolic Ca^2+^ rise in response to different degree of stretch. Change in cytosolic Ca^2+^ was expressed as F_x_/F_0_, where F_x_ is that real-time fluo-4/AM signal and F_0_ is the baseline fluo-4/AM signal before stretch. Mean ± s.e.m. (*n* = 5). ^*^p < 0.05, ^***^p < 0.01 as compared to the corresponding control, by pairwise *t*-test.

Cultured aortic depressor or carotid sinus neurons were subjected to 10, 20, or 30% of uniaxial stretch by STREX Cell Stretching System, attempting to mimic the blood vessel stretching under blood pressure. The results showed that a uniaxial stretch of 20% induced a marked increase in cytosolic Ca^2+^ fluorescence in aortic baroreceptor neurons but not in carotid baroreceptor neurons (Figures 4A–C). Furthermore, the magnitude of Ca^2+^ fluorescence rises in response to 30% uniaxial stretch was much higher in aortic baroreceptor neurons than in carotid baroreceptor neurons (Figures 4A–C).

### Baroreceptor nerve activity *in vivo*

**Figure 5:**
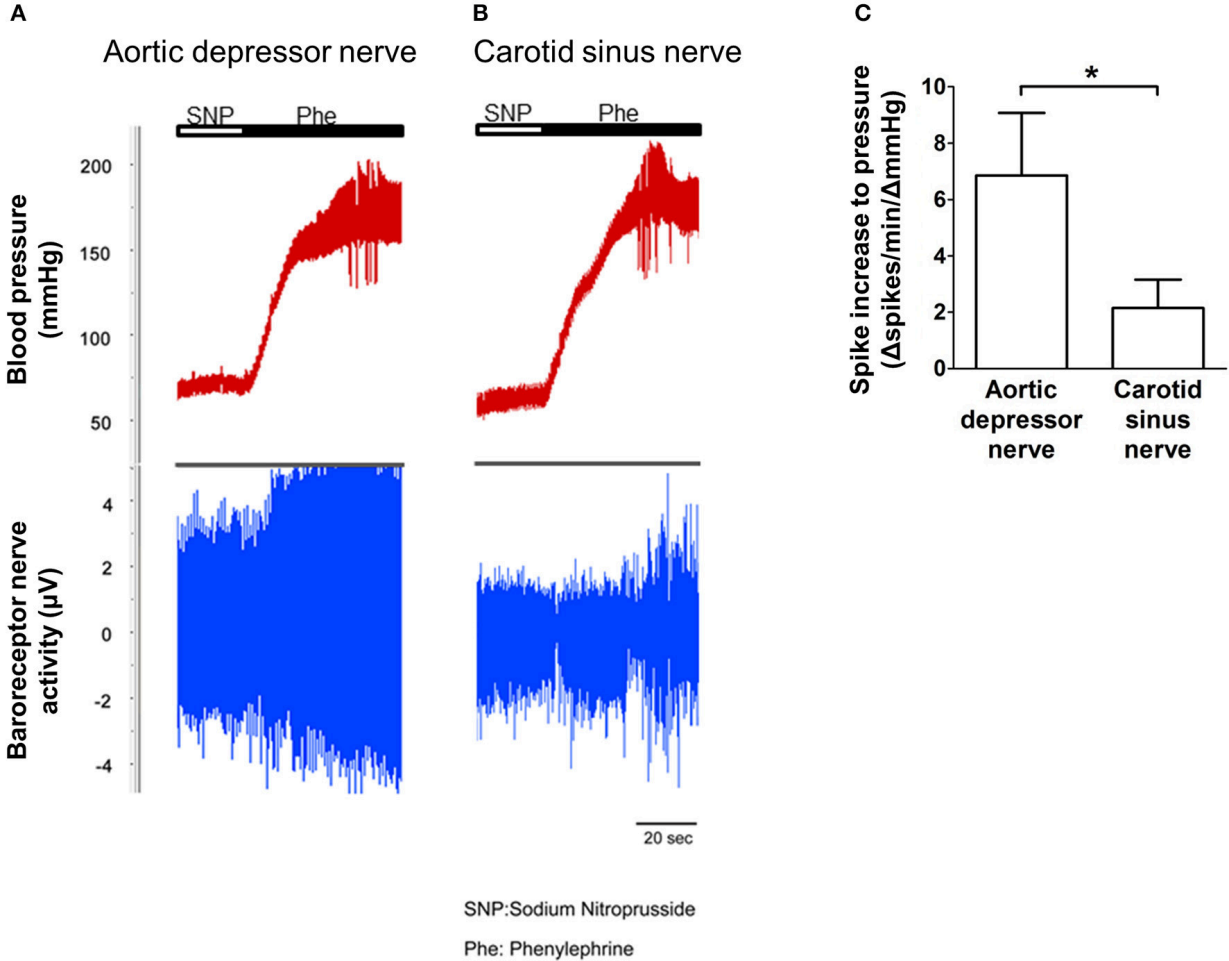
**Baroreceptor nerve activity in rat *in vivo*. (A,B)** Representative traces showing the change in aortic depressor nerve **(A)** and carotid sinus nerve **(B)** activity upon blood pressure elevation. In both **(A,B)**, upper panel was the change in blood pressure before and after Phe application as indicated by the horizontal bars. Lower panel was the corresponding change in nerve activity. **(C)** Summarized data showing the change in nerve activity per unit of blood pressure change. Mean ± s.e.m. (*n* = 8). ^*^p < 0.05 as compared to the control in aortic baroreceptors, by pairwise *t*-test.

The nerve activity of the two baroreceptors was investigated *in vivo*. Phenylephrine was injected intravenously. The changes in blood pressure upon phenylephrine application are shown in the upper panel of Figures 5A,B. The subsequence changes in baroreceptor nerve activity are shown in the lower panel (Figures 5A,B). The pressure-induced nerve activity was much higher in aortic depressor nerve than in carotid sinus nerve (Figures 5A,B). The change in spike frequency per mmHg increase is summarized in Figure 5C. The result indicated that the aortic baroreceptors are more sensitive to hydrostatic pressure than the carotid baroreceptors *in vivo*.

### Expression of TRP channels

**Figure 6:**
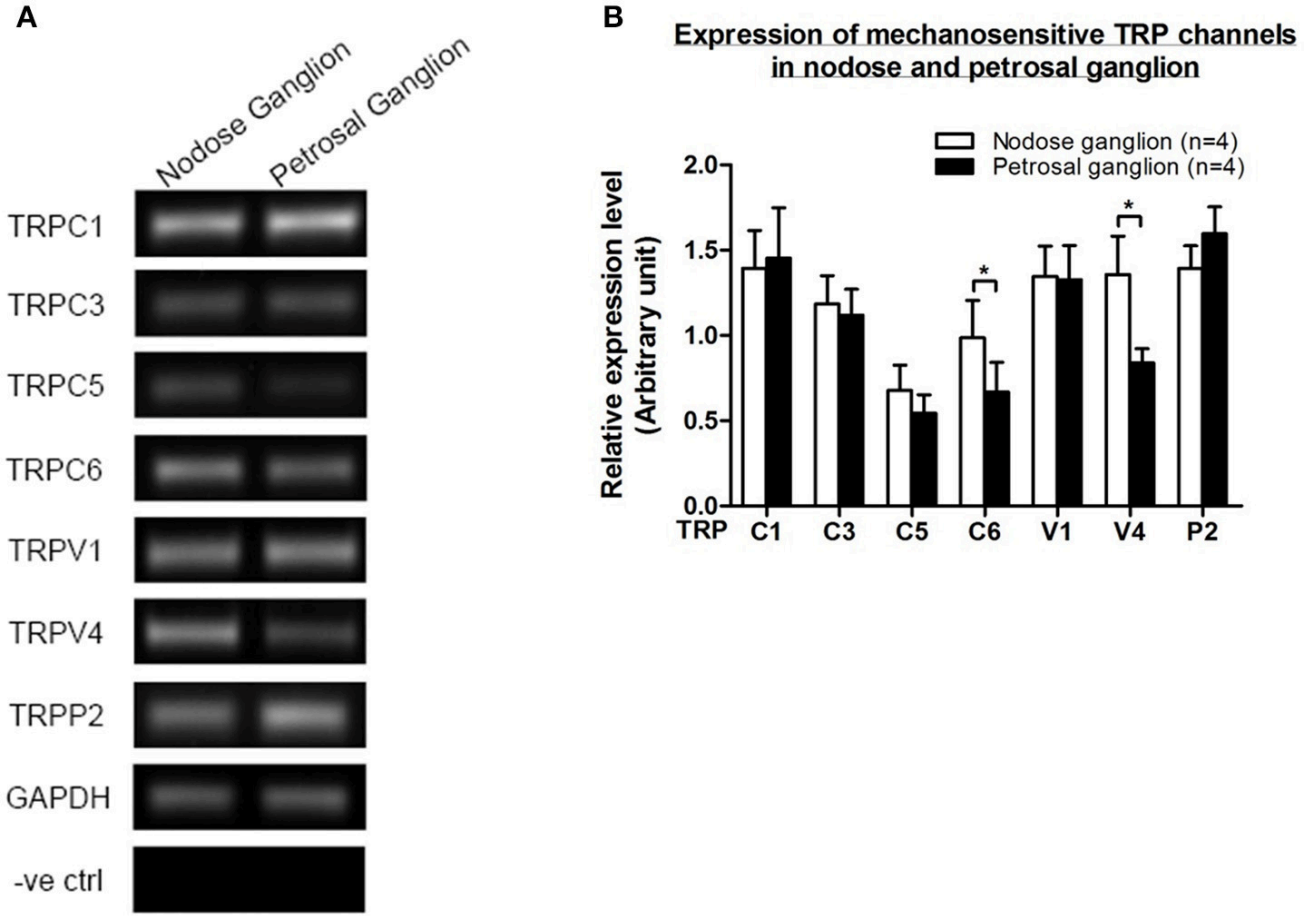
**Expression of mechanosensitive TRP channels in nodose and petrosal ganglion. (A)** Representative gel images comparing the mRNA expression level of mechanosensitive TRP channels, (TRPC1, -C3, -C5, -C6, -V1, -V4, -P2) between nodose and petrosal ganglion neurons. Semi-quantitative RT-PCR analysis was used to compare the mRNA expression levels of several TRP channels relative to GAPDH mRNA expression. The results of 9 experiments were summarized in **(B)**. Values were in mean ± sem. ^*^p < 0.05, by pairwise *t*-test.

Several TRP channels have been reported to be mechano-sensitive, including TRPC1, -C3, -C5, -C6, -V1, -V4, -P2 (O'Neil and Heller, 2005; Christensen and Corey, 2007; Pedersen and Nilius, 2007; Patel et al., 2010; Lau et al., 2016). Here, we investigated the possible role of these mechanosensitive TRP channels in relationship to aortic and carotid baroreceptor sensitivity. The mRNA expressions of these channels in nodose and petrosal ganglion were compared. It was found that the expressions of TRPV4 and TRPC6 were higher in nodose ganglion as compared to petrosal ganglion (Figure 6). It is possible that the higher expression of mechanosensitive channels in nodose ganglion may contribute to the higher sensitivity.

## Discussion

In the present study, the mechanosensitive properties of aortic and carotid baroreceptors were compared. Whole-cell patch clamp studies showed that, compared to the carotid sinus neurons, a higher percentage of aortic depressor neurons were stretch-sensitive. Furthermore, the pressure threshold that could initiate action potential firings was found to be lower in the aortic baroreceptor neurons than in the carotid baroreceptor neurons. Uniaxial stretch-induced cytosolic Ca^2+^ rise was also compared between the aortic depressor and carotid sinus neurons. The magnitude of the cytosolic Ca^2+^ rise in response to the uniaxial stretch was much higher in aortic baroreceptor neurons than in carotid baroreceptor neurons. Aortic baroreceptor neurons also displayed a lower threshold of cytosolic Ca^2+^ responses to uniaxial stretch. In another series of experiments, we found that pressure-induced increase in action potential firings was higher in aortic depressor nerve than in the carotid sinus nerve. Taken together, our data provide strong evidence for the aortic baroreceptors being more sensitive to blood pressure than carotid baroreceptors.

Previously, much effort has been taken to understand the difference in sensitivities between the two baroreceptors. However, the results are controversial. Some reports concluded that the aortic baroreceptors are more sensitive to pressure change (Glick and Covell, 1968; Fan et al., 1996; Pickering et al., 2008), whereas others stated that the carotid baroreceptors are more sensitive to pressure change (Donald and Edis, 1971; Fadel et al., 2003). However, almost all of these previous studies involved highly complicated surgical procedures and ultimately utilized baroreflex responses, such as heart rate and renal sympathetic output, as the end points for baroreceptor sensitivity assay. Complicated surgical procedures are prone to introduce experimental errors, which may result in controversial conclusions. Furthermore, animal species-dependent variation in baroreceptor sensitivity is also possible. Moreover, the baroreflex response is not only influenced by baroreceptor sensitivity, but also by multiple other factors including nerve conduction, central mediation, blood vessel contractility and heart function. Therefore, the evidence from previous studies is indirect and hardly ideal. Up to the present, there is a lack of direct studies comparing the pressure sensitivity of aortic vs. carotid baroreceptor neurons. In this study, the mechanosensitivity was studied directly in baroreceptor neurons. The data strongly suggest that the aortic baroreceptors are more sensitive to pressure than the carotid baroreceptors. This information may have important physiological relevance in blood pressure regulation.

It is unclear what mechanisms underlie the difference in pressure sensitivity between aortic and carotid baroreceptors. We compared the expression level of several mechanosensitive TRP channels (Figure 6) (O'Neil and Heller, 2005; Christensen and Corey, 2007; Pedersen and Nilius, 2007; Patel et al., 2010). Our results showed that aortic baroreceptor neurons have a higher expression of several mechanosensitive TRP channels including TRPV4 and TRPC6. It is possible that higher expression of TRPV4 and/or TRPC6 may contribute to the high pressure sensitivity of aortic baroreceptors. Further studies are needed to verify the role of these ion channels.

It was previously reported that nodose ganglion and petrosal ganglion contain two different types of neurons (Belmonte and Gallego, 1983). One of them gives continuous action potentials upon electrical stimulation, while the other just gives a single action potential upon stimulation. The authors assigned the former as the pressure-sensitive neurons, and assigned the latter as the chemo-sensitive neurons. Our results indicate that it may not be appropriate to assign the pressure-sensitive neurons and pressure-insensitive neurons just based on whether they fire a single action potential or continuous action potentials in response to the depolarizing current. In fact, a large percentage of cells that fire a single action potential in response to electrical stimulation are also sensitive to pressure, and thus they belong to pressure-sensing baroreceptor neurons (Figure 2 and Table 1).

In summary, the present study provides evidence that the aortic baroreceptors are more sensitive to pressure than the carotid baroreceptors and adds new information to the understanding of the basic physiology.

## Author contributions

EL and XY design the experiments and draft the manuscript. EL, CL, and YY perform the experiments. XY, YH, AM, and LJ revised the manuscript. All authors approved the final version of the manuscript.

## Funding

This work was supported by grants from the Hong Kong Research Grant Committee CUHK478710, CUHK478413, AoE/M-05/12, TBRS/T13-706/11, China National Science Foundation Grant 31470912 and RGC-NSFC Joint Grant N_CUHK439/13.

### Conflict of interest statement

The authors declare that the research was conducted in the absence of any commercial or financial relationships that could be construed as a potential conflict of interest.
